# An Effective Hybrid Cuckoo Search Algorithm with Improved Shuffled Frog Leaping Algorithm for 0-1 Knapsack Problems

**DOI:** 10.1155/2014/857254

**Published:** 2014-10-22

**Authors:** Yanhong Feng, Gai-Ge Wang, Qingjiang Feng, Xiang-Jun Zhao

**Affiliations:** ^1^School of Information Engineering, Shijiazhuang University of Economics, Shijiazhuang 050031, China; ^2^School of Computer Science and Technology, Jiangsu Normal University, Xuzhou, Jiangsu 221116, China; ^3^School of Mathematical Science, Kaili University, Kaili, Guizhou 556011, China

## Abstract

An effective hybrid cuckoo search algorithm (CS) with improved shuffled frog-leaping algorithm (ISFLA) is put forward for solving 0-1 knapsack problem. First of all, with the framework of SFLA, an improved frog-leap operator is designed with the effect of the global optimal information on the frog leaping and information exchange between frog individuals combined with genetic mutation with a small probability. Subsequently, in order to improve the convergence speed and enhance the exploitation ability, a novel CS model is proposed with considering the specific advantages of Lévy flights and frog-leap operator. Furthermore, the greedy transform method is used to repair the infeasible solution and optimize the feasible solution. Finally, numerical simulations are carried out on six different types of 0-1 knapsack instances, and the comparative results have shown the effectiveness of the proposed algorithm and its ability to achieve good quality solutions, which outperforms the binary cuckoo search, the binary differential evolution, and the genetic algorithm.

## 1. Introduction

The application of nature-inspired metaheuristic algorithms to computational optimization is a growing trend [1]. Many hugely popular algorithms, including differential evolution (DE) [2, 3], harmony search (HS) [4, 5], krill herd algorithm (KH) [6–13], animal migration optimization (AMO) [14], grey wolf optimizer (GWO) [15], biogeography-based optimization (BBO) [16, 17], gravitational search algorithm (GSA) [18], and bat algorithm (BA) [19, 20], perform powerfully and efficiently in solving diverse optimization problems. Many metaheuristic algorithms have been applied to solve knapsack problems, such as evolutionary algorithms (EA) [21], HS [22], chemical reaction optimization (CRO) [23], cuckoo search (CS) [24–26], and shuffled frog-leaping algorithm (SFLA) [27]. To better understand swarm intelligence please refer to [28].

In 2003, Eusuff and Lansey firstly proposed a novel metaheuristic optimization method: SFLA, which mimics a group of frogs to search for the location that has the maximum amount of available food. Due to the distinguished benefit of its fast convergence speed, SFLA has been successfully applied to handle many complicated optimization problems, such as water resource distribution [29], function optimization [30], and resource-constrained project scheduling problem [31].

CS, a nature-inspired metaheuristic algorithm, is originally proposed by Yang and Deb in 2009 [32], which showed some promising efficiency for global optimization. Owing to the outstanding characteristics such as fewer parameters, easy implementation, and rapid convergence, it is becoming a new research hotspot in swarm intelligence. Gandomi et al. [33] first verified structural engineering optimization problems with CS algorithm. Walton et al. [34] proposed an improved cuckoo search algorithm which involved the addition of information exchange between the best solutions and tested their performance with a set of benchmark functions. Recently, the hybrid algorithms that combined CS with other methods have been proposed and have become a hot topic studied by people, such as CS combined with a fuzzy system [35], a DE [36], wind driven optimization (WDO) [37], artificial neural network (ANN) [38], and genetic algorithm (GA) [39]. For details, see [40].

In 2011, Layeb [25] developed a variant of cuckoo search in combination with quantum-based approach to solve knapsack problems efficiently. Subsequently, Gherboudj et al. [24] utilized purely binary cuckoo search to tackle knapsack problems. A few scholars consider binary-coded CS and its performance need to further improve so as to further expand its fields of application. In addition, despite successful application to the solution of 0-1 knapsack problem by many methods, in fact, it is still a very active research area, because many existing algorithms do not cope well with some new and more intractable 0-1 knapsack problems hidden in the real world. Further, most of recently proposed algorithms focused on solving 0-1 knapsack problems with low dimension and medium dimension, but 0-1 knapsack problems with high dimension are involved little and the results are not highly satisfactory. What is more, the correlation between the weight and the value of the items may not be more concerned. This necessitates new techniques to be developed.

Therefore, in this work, we propose a hybrid CS algorithm with improved SFLA (CSISFLA) for solving 0-1 knapsack problem. To verify effectiveness of our proposed method, a large number of experiments on 0-1 knapsack problem are conducted and the experimental results show that the proposed hybrid metaheuristic method can reach the required optima more effectively than CS, DE, and GA even in some cases when the problem to be solved is too complicated and complex.

The rest of the paper is organized as follows.  Section 2 introduces the preliminary knowledge of CS, SFLA algorithm, and the mathematical model of 0-1 KP problem. Then, our proposed CSISFLA for 0-1 KP problems is presented in Section 3. A series of simulation experiments are conducted in Section 4. Some conclusions and comments are made for further research in Section 5.

## 2. Review of the Related Work

In this section, the model of 0-1 knapsack problem and the basic CS and SFLA are introduced briefly.

### 2.1. 0-1 Knapsack Problem

The 0-1 knapsack problem, denoted by KP, is a classical optimization problem and it has high theoretical and practical value. Many practical applications can be formulated as a KP, such as cutting stock problems, portfolio optimization, scheduling problems, and cryptography. This problem has been proven to be a NP-hard problem; hence, it cannot be solved in a polynomial time unless *P* = *NP* [44].

The 0-1 knapsack problem can be stated as follows:

(1) Maximize f(x)=∑j=1npjxjsubject to ∑j=1nwjxj≤c, xj=0  or  1, j=1,…,n,

where *n* is the number of items; *w*_*j*_ and *p*_*j*_ represent the weight and profit of item* j*, respectively. The objective is to select some items so that the total weight does not exceed a given capacity* c*, while the total profit is maximized. The binary decision variable *x*_*i*_, with *x*_*i*_ = 1 if item *i* is selected, and *x*_*i*_ = 0 otherwise is used.

### 2.2. Cuckoo Search

CS is a relatively new metaheuristic algorithm for solving global optimization problems, which is based on the obligate brood parasitic behavior of some cuckoo species. In addition, this algorithm is enhanced by the so-called Lévy flights rather than by simple isotropic random walks.

For simplicity, Yang and Deb used the following three approximate rules [32, 45]:

1. each cuckoo lays only one egg at a time and dumps its egg in a randomly chosen nest;
2. the best nests with high-quality eggs will be carried over to the next generations;
3. the number of available host nests is fixed, and the egg laid by the host bird with a probability *p*_*a*_ ∈ [0,1]. In this case, the host bird can either throw the egg away or simply abandon the nest and build a completely new nest.

The last assumption can be approximated by a fraction *p*_*a*_ of the *n* host nests which are replaced by new nests (with new random solutions).

New solution **X**_*i*_^(*t*+1)^ is generated as (2) by using a Lévy flight [32]. Lévy flights essentially provide a random walk while their random steps followed a Lévy distribution for large steps which has an infinite variance with an infinite mean. Here the steps essentially form a random walk process with a power-law step-length distribution with a heavy tail as (3):

(2) $$\begin{array}{c} X_{i}^{\left(t+1\right)}=X_{i}^{\left(t\right)}+\alpha ⊕\text{Levy}\left(\lambda \right), \end{array}$$

(3) $$\begin{array}{c} \text{Levy}\left(\lambda \right)\sim u=t^{-\lambda }, \end{array}$$

where *α* > 0 is the step size scaling factor. Generally, we take *α* = *O*  (1). The product ⊕ means entry-wise multiplications.

### 2.3. Shuffled Frog-Leaping Algorithm

The SFLA is a metaheuristic optimization method that imitates the memetic evolution of a group of frogs while casting about for the location that has the maximum amount of available food [46]. SFLA, originally developed by Eusuff and Lansey in 2003, can be applied to handle many complicated optimization problems. In virtue of the beneficial combination of the genetic-based memetic algorithm (MA) and the social behavior-based PSO algorithm, the SFLA has the advantages of global information exchange and local fine search. In SFLA, all virtual frogs are assigned to disjoint subsets of the whole population called memeplex. The different memeplexes are regarded as different cultures of frogs and independently perform local search. The individual frogs in each memeplex have ideas that can be effected by the ideas of other frogs and evolve by means of memetic evolution. After a defined number of memetic evolution steps, ideas are transferred among memeplexes in a shuffling process. The local search and the shuffling processes continue until defined convergence criteria are satisfied [47].

In the SFLA, the initial population *P* is partitioned into *M* memeplexes, each containing *N* frogs (*P* = *M* × *N*). In this process, the *i*th goes to the* j*th memeplex where *j* = *i* mod* M* (memeplex numbered from 0). The procedure of evolution of individual frogs contains three frog leapings. The position update is as follows.

Firstly, the new position of the frog individual is calculated by

(4) $$\begin{array}{c} Y=X+r_{1}\times \left(B_{k}-W_{k}\right). \end{array}$$

If the new position *Y* is better than the original position *X*, replace *X* with *Y*; else, another new position of this frog will perform in which the global optimal individual *B*_*g*_ replaces the best individual of* k*th memeplex *B*_*k*_ with the following leaping step size:

(5) $$\begin{array}{c} Y=X+r_{2}\times \left(B_{g}-W_{k}\right). \end{array}$$

If nonimprovement becomes possible in this case, the new frog is replaced by a randomly generated frog; else replace *X* with* Y*:

(6) $$\begin{array}{c} Y=L+r_{3}\times \left(U-L\right). \end{array}$$

Here,* Y* is an update of frog's position in one leap. *r*_1_, *r*_2_, and *r*_3_ are random numbers uniformly distributed in [0,1]. *B*_*k*_ and *W*_*k*_ are the best and the worst individual of the* k*th memeplex, respectively. *B*_*g*_ is the best individual in the whole population.* U*,* L* is the maximum and minimum allowed change of frog's position in one leap.

## 3. Hybrid CS with ISFLA for 0-1 Knapsack Problems

In this section, we will propose a hybrid metaheuristic algorithm integrating cuckoo search and improved shuffled frog-leaping algorithm (CSISFLA) for solving 0-1 knapsack problem. First, the hybrid encoding scheme and repair operator will be introduced. And then improved frog-leaping algorithm along with the framework of proposed CSISFLA will be presented.

### 3.1. Encoding Scheme

As far as we know, the standard CS algorithm can solve the optimization problems in continuous space. Additionally, the operation of the original CS algorithm is closed to the set of real number, but it does not have the closure property in the binary set {0,1}. Based on above analysis, we utilize hybrid encoding scheme [26] and each cuckoo individual is represented by two tuples 〈*x*_*j*_, *b*_*j*_〉 (*j* = 1,2,…, *d*), where *x*_*j*_ works in the auxiliary search space and *b*_*j*_ performs in the solution space accordingly and *d* is the dimensionality of solution. Further, Sigmoid function is adopted to transform a real-coded vector **X**_*i*_ = (*x*_1_,*x*_2_,…,*x*_*d*_)^T^ ∈ [−3.0,3.0]^*d*^ to binary vector **B**_*i*_ = (*b*_1_,*b*_2_,…,*b*_*d*_)^T^ ∈ {0,1}^*d*^. The procedure works as follows:

(7) $$\begin{array}{c} b_{i}=\{\begin{array}{cc} 1, & \text{if}\;\;\text{Sig}\left(x_{i}\right)\geq 0.5,\\ 0, & \text{else}, \end{array} \end{array}$$

where Sig(*x*) = 1/(1 + *e* ^−*x*^) is Sigmoid function.

The encoding scheme of the population is depicted in Table 1.

### 3.2. Repair Operator

After evolving a generation, the feasibility of all the generated solutions is taken into consideration. That is, to say, the individuals could be illegal because of violating the constraint conditions. Therefore, a repair procedure is essential to construct illegal individuals. In this paper, an effective greedy transform method (GTM) is introduced to solve this problem [26, 48]. It cannot only effectively repair the infeasible solution but also can optimize the feasible solution.

This GTM consists of two phases. The first phase, called repairing phase (RP), checks each solution in order of decreasing *p*_*i*_/*w*_*i*_ and confirms the variable value of one as long as feasibility is not violated. The second phase, called optimizing phase (OP), changes the remaining variable from zero to one until the feasibility is violated. The primary aim of the OP is to transform an abnormal chromosome coding into a normal chromosome, while the RP is to achieve the best chromosome coding.

### 3.3. Improved Shuffled Frog-Leaping Algorithm

In the evolution of SFLA, new individual is only affected by local optimal individual and the global optimal during the first two frog leapings, respectively. That is to say, there is a lack of information exchange between individuals and memeplexes. In addition, the use of the worst individual is not conducive to quickly obtain the better individuals and quick convergence. When the quality of the solution has not been improved after the first two frog leapings, the SFLA randomly generates a new individual without restriction to replace original individual, which will result in the loss of some valuable information of the superior individual to some extent. Therefore, in order to make up for the defect of the SFLA, an improved shuffled frog-leaping algorithm (ISFLA) is carefully designed and then embedded in the CSISFLA. Compared with SFLA, there are three main improvements.

The first slight improvement is that we get rid of sorting of the items according to the fitness value which will decrease in time cost.

The second improvement is that we adopt a new frog individual position update formula instead of the first two frog leapings. The idea is inspired by the DE/Best/1/Bin in DE algorithm. Similarly, each frog individual *i* is represented as a solution **X**_*i*_ and then the new solution *Y* is given by

(8) $$\begin{array}{c} Y=B_{g}\pm r_{2}\times \left(B_{k}-X_{p1}\right), \end{array}$$

where *B*_*g*_ is the current global best solution found so far. *B*_*k*_ is the best solution of the* k*th memeplex. *X*_*p*1_ is an individual of random selection with index of *p*1 ≠ *i* and *r*_2_ is random number uniformly distributed in [0,1]. In particular the plus or minus signs are selected with certain probability. The main purpose of improvement in (8) is to quicken convergence rate.

The third improvement is to randomly generate new individuals with certain probability instead of unconditional generating new individuals, which takes into consideration the retention of the better individuals in the population.

The main step of ISFLA is given in Algorithm 1. In Algorithm 1,* P* is the size of the population.* M* is the number of memeplex.* D* is the dimension of decision variables. And *r*_1_ is a random real number uniformly distributed in (0, 1). And *r*_2_, *r*_3_, *r*_4_, and *p*_*m*_ are all* D*-dimensional random vectors and each dimension is uniformly distributed in (0, 1). In particular, *p*_*m*_ is called probability of mutation which controls the probability of individual random initialization.

### 3.4. The Frame of CSISFLA

**Figure 1:**
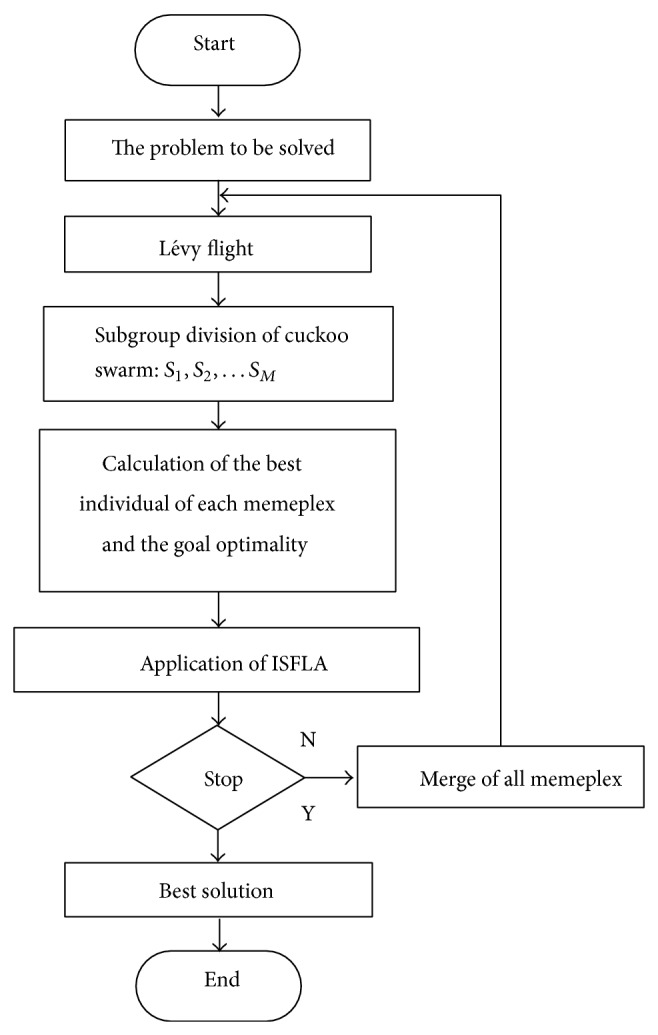
The architecture of CSISFLA algorithm.

In this section, we will demonstrate how we combine the well-designed ISFLA with Lévy flights to form an effective CSISFLA. The proposed algorithm does not change the main search mechanism of CS and SFLA. In the iterative process of the whole population, Lévy flights are firstly performed and then frog-leaping operator is adopted in each memeplex. Therefore, the strong exploration abilities in global area of the original CS and the exploitation abilities in local region of ISFLA can be fully developed. The CSISFLA architecture is explained in Figure 1.

### 3.5. CSISFLA Algorithm for 0-1 Knapsack Problems

Through the design above carefully, the pseudocode of CSISFLA for 0-1 knapsack problems is described as follows (see Algorithm 2). It can be analyzed that there are essentially three main processes besides the initialization process. Firstly, Lévy flights are executed to get a cuckoo randomly or generate a solution. The random walk via Lévy flights is much more efficient in exploring the search space owing to its longer step length. In addition, some of the new solutions are generated by Lévy flights around the best solution, which can speed up the local search. Then ISFLA is performed in order to exploit the local area efficiently. Here, we regard the frog-leaping process as the process of cuckoo laying egg in a nest. The new nest generated with a probability *p*_*m*_ is far enough from the current best solution, which enables CSISFLA to avoid being trapped into local optimum. Finally, when an infeasible solution is generated, a repair procedure is adopted to keep feasibility and, moreover, optimize the feasible solution. Since the algorithm can well balance the exploitation and exploration, it expects to obtain solutions with satisfactory quality.

### 3.6. Algorithm Complexity

CSISFLA is composed of three stages: the sorting by value-to-weight ratio, the initialization, and the iterative search. The quick sorting has time complexity *O* (*P*log⁡ (*P*)). The generation of the initial cuckoo nests has time complexity *O* (*P* × *D*). The iterative search consists of four steps (comment statements in Algorithm 2), and so forth, the Lévy flight, the first frog leaping, generate new individual and random selection which costs the same time *O* (*D*). In summary, the overall complexity of the proposed CSISFLA is *O* (*P*log⁡ (*P*)) + *O* (*P* × *D*) + *O* (*D*) = *O* (*P*log⁡ (*P*)) + *O* (*P* × *D*). It does not change compared with the original CS algorithm.

## 4. Simulation Experiments

### 4.1. Experimental Data Set

In existent researching files, cases studies and research of knapsack problems are about small-scale to moderate-scale problems. However, in real-world applications, problems are typically large-scale with thousands or even millions of design variables. In addition, the complexity of KP problem is greatly affected by the correlation between profits and weights [49–51]. However, few scholars pay close attention to the correlation between the weight and the value of the items. To test the validity of the algorithm for different types of instances, we adopt uncorrelated, weakly correlated, strongly correlated, multiple strongly correlated, profit ceiling, and circle data sets with different dimension. The problems are described as follows:

- uncorrelated instances: the weights *w*_*j*_ and the profits *p*_*j*_ are random integers uniformly distributed in [10,100];
- weakly correlated instances: the weights *w*_*j*_ are random integers uniformly distributed in [10,100], and the profits *p*_*j*_ are random integer uniformly distributed in [*w*_*j*_ − 10, *w*_*j*_ + 10];
- strongly correlated instances: the weights *w*_*j*_ are random integers uniformly distributed in [10,100] and the profits *p*_*j*_ are set to *w*_*j*_ + 10;
- multiple strongly correlated instances: the weights *w*_*j*_ are randomly distributed in [10,100]. If the weight *w*_*j*_ is divisible by 6, then we set the *p*_*j*_ = *w*_*j*_ + 30 otherwise set it to *p*_*j*_ = *w*_*j*_ + 20;
- profit ceiling instances: the weights *w*_*j*_ are randomly distributed in [10,100] and the profits *p*_*j*_ are set to *p*_*j*_ = 3⌈*w*_*j*_/3⌉;
- circle instances: the weights *w*_*j*_ are randomly distributed in [10,100] and the profits *p*_*j*_ are set to $p_{j}=d\sqrt{4R^{2}-{\left(w_{j}-2R\right)}^{2}}$. Choosing *d* = 2/3, *R* = 10.

For each data set, we set the value of the capacity. Consider *c* = 0.75∑_*j*=1_^*n*^ *w*_*j*_.

**Figure 2:**
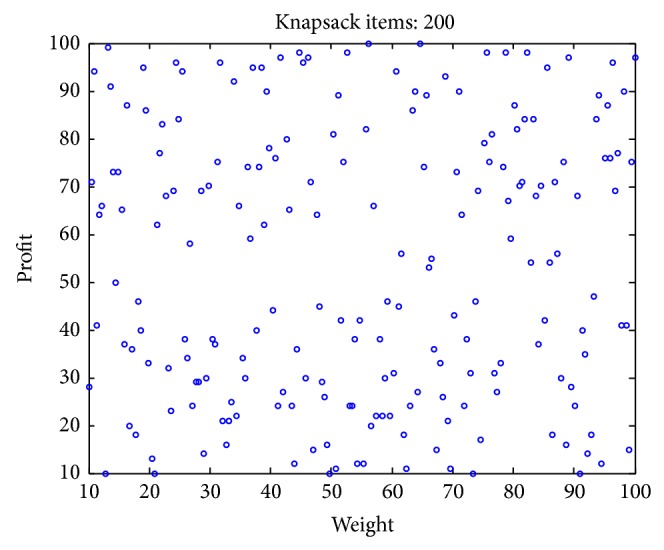
Uncorrelated items.

**Figure 3:**
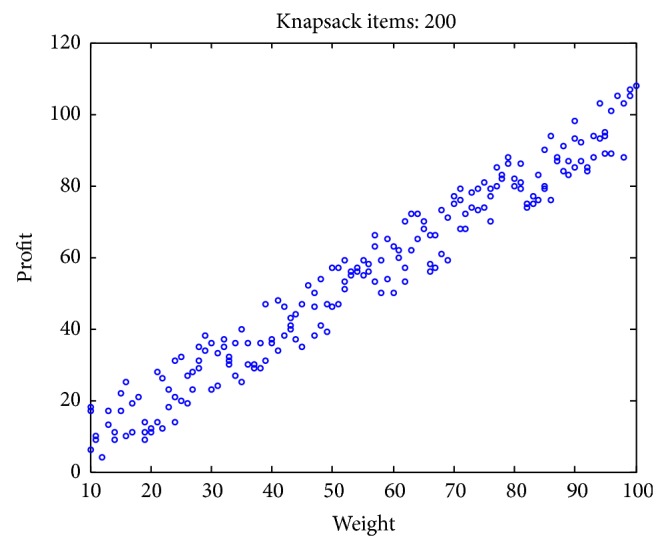
Weakly correlated items.

**Figure 4:**
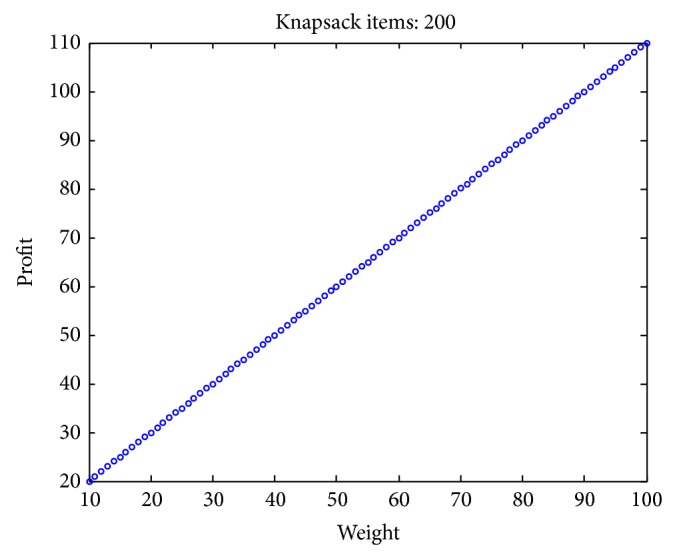
Strongly correlated items.

**Figure 5:**
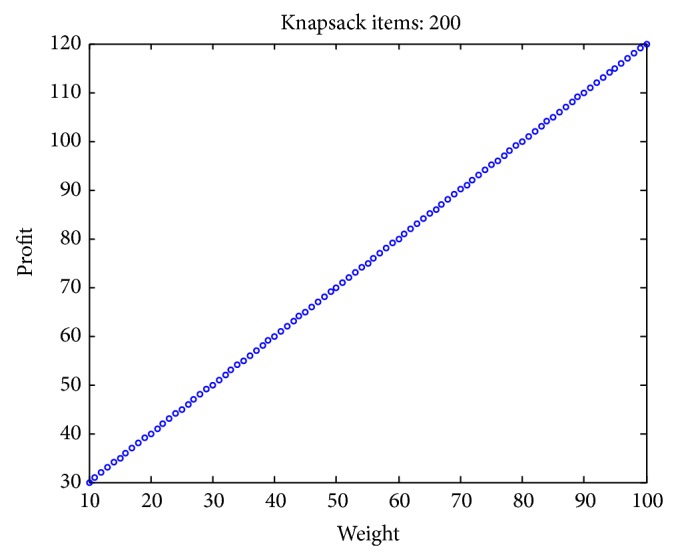
Multiple strongly correlated items.

**Figure 6:**
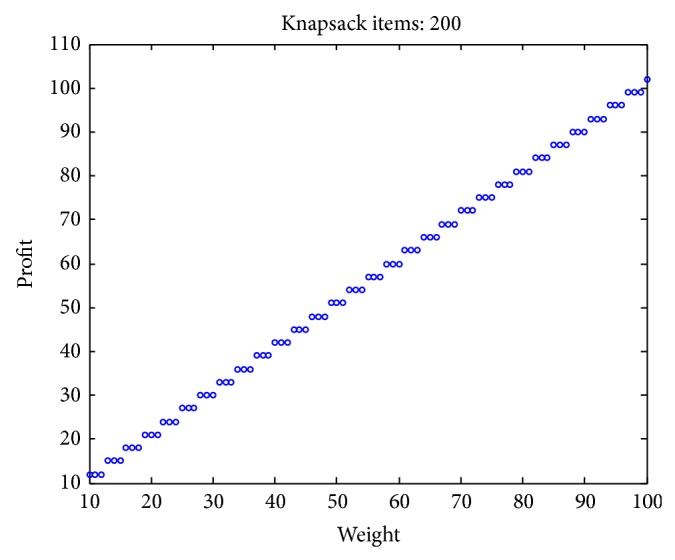
Profit ceiling items.

**Figure 7:**
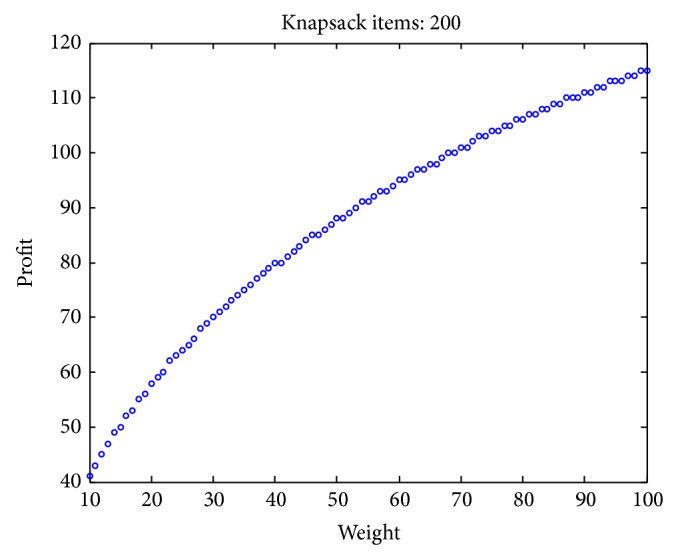
Circle items.

Figures 2, 3, 4, 5, 6, and 7 illustrate six types of instances of 200 items, respectively.

The KP instances in this study are described in Table 2.

### 4.2. The Selection on the Value of *M* and* N*

The CSISFLA has some control parameters that affect its performance. In our experiments, we investigate thoroughly the number of subgroups *M* and the number of individuals in each subgroup *N*. The below three test instances are used to study the effect of *M* and *N* on the performance of the proposed algorithm. Firstly,* M* is set to 2, and then three levels of 10, 15, and 20 are considered for* N* (accordingly, the size of population is 2 × 10, 2 × 15, and 2 × 20). Secondly, a fixed individual number of each subgroup is 10, and the value of *M* is 2, 3, and 4, respectively. Results are summarized in Table 3.

As expected, with the increase of the individual number in the population, it is an inevitable consequence that there are more opportunities to obtain the optimal solution. This issue can be indicated by bold data in Table 3. In order to get a reasonable quality under the condition of inexpensive computational costs, we use *N* = 10 and *M* = 4 in the rest experiments.

### 4.3. The Selection on the Value of *p*_*m*_

In this subsection, the effect of *p*_*m*_ on the performance of the CSISFLA is carefully investigated. We select two uncorrelated instances (KP_1_, KP_2_) and two weakly correlated instances (KP_8_, KP_9_) as the test instances for parameter setting experiment of *p*_*m*_. For each instance, every test is run 30 times. We use *N* = 10, *M* = 4, and the maximum time of iterations is set to 5 seconds.  Table 4 gives the optimization results of the CSISFLA using different values for *p*_*m*_.

From the results of Table 4, it is not difficult to observe that the probability of mutation with 0.05 ≤ *p*_*m*_ ≤ 0.4 is more suitable for all test instances which can be seen from data in bold in Table 3. In addition, the optimal solution dwindles steadily with the change of *p*_*m*_ from 0.5 to 1.0 and the worst results of four evaluation criteria are obtained when *p*_*m*_ = 1. Similarly, the performance of the CSISFLA is also poor when *p*_*m*_ is 0. As we have expected, 0 means that the position update in memeplex is completed entirely by the first Leapfrog, which cannot effectively ensure the diversity of the entire population, leading to the CSISFLA more easily fall into the local optimum, and 1 means that new individuals randomly generated without any restrictions which results in slow convergence. Generally speaking, using a small value of *p*_*m*_ is beneficial to strengthen the convergence ability and stability of the CSISFLA. The performance of the algorithm is the best when *p*_*m*_ = 0.15, so we will set *p*_*m*_ = 0.15 for the following experiments.

### 4.4. Experimental Setup and Parameters Setting

In this paper, in order to test the optimization ability of CSISFLA and further investigate effectiveness of the algorithms for different types of instance, we adopt a set of 34 knapsack problems (KP_1_–KP_34_). We compared the performance of CSISFLA with (a) GA, (b) DE, and (c) classical CS. In the experiments, the parameters setting are shown in Table 5.

In order to make a fair comparison, all computational experiments are conducted with Visual C++ 6.0. The test environment is set up on a PC with AMD Athlon(tm) II X2 250 Processor 3.01 GHz, 1.75 G RAM, running on Windows XP. The experiment on each instance was repeated 30 times independently. Further, best solution, worst solution, mean, median, and standard deviation (STD) for all the solutions are given in related tables. In addition, the maximum run-time was set to 5 seconds for the instances with dimension less than 500, and it was set to 8 seconds for other instances.

### 4.5. The Experimental Results and Analysis

**Figure 8:**
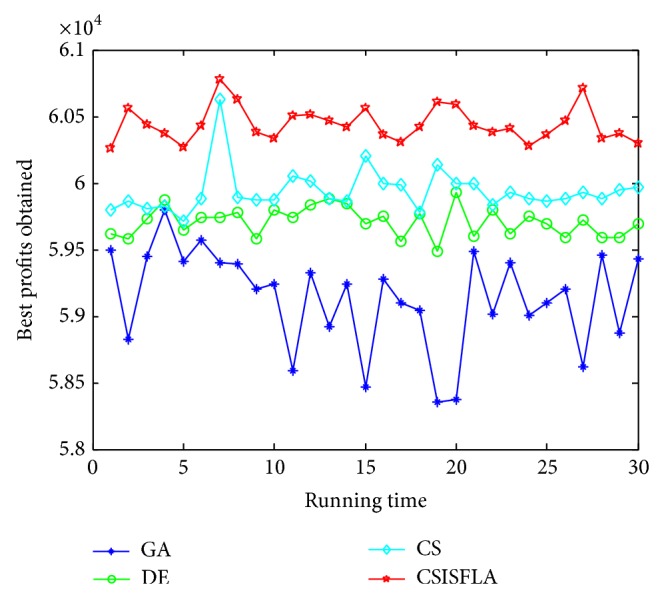
The best profits obtained in 30 runs for KP_7_.

**Figure 9:**
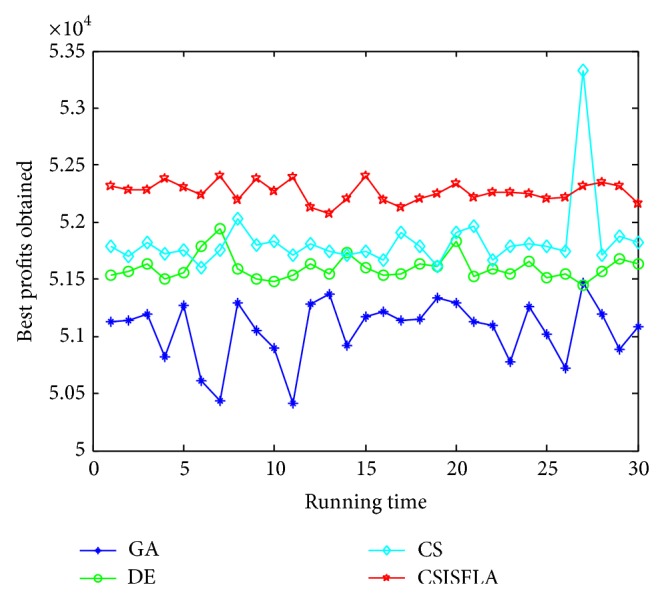
The best profits obtained in 30 runs for KP_14_.

**Figure 10:**
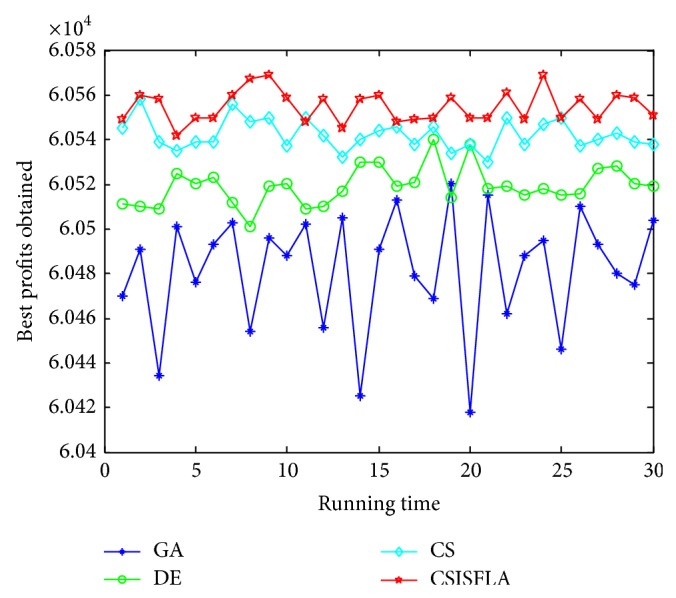
The best profits obtained in 30 runs for KP_19_.

**Figure 11:**
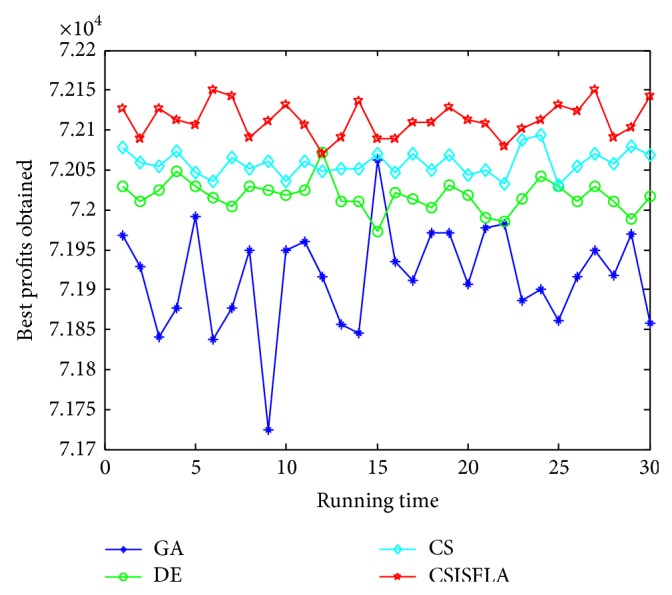
The best profits obtained in 30 runs for KP_24_.

**Figure 12:**
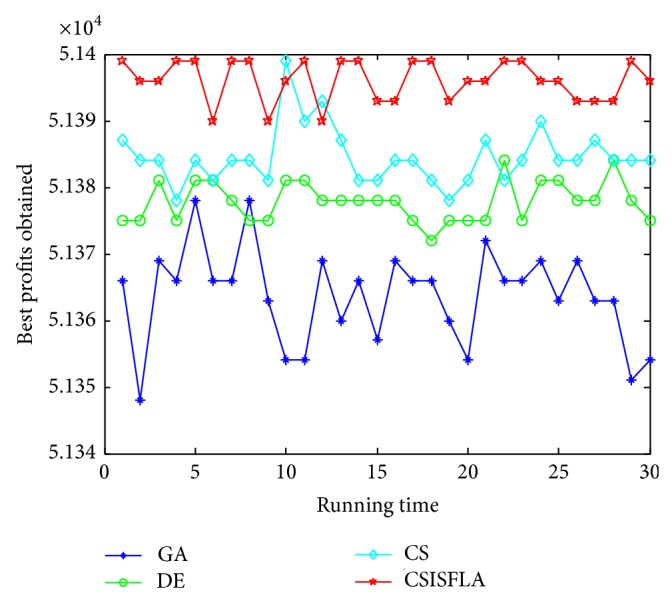
The best profits obtained in 30 runs for KP_29_.

**Figure 13:**
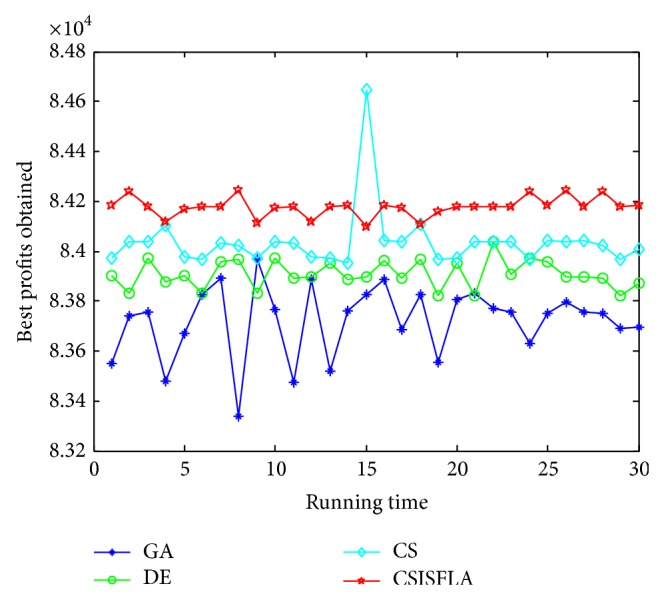
The best profits obtained in 30 runs for KP_34_.

**Figure 14:**
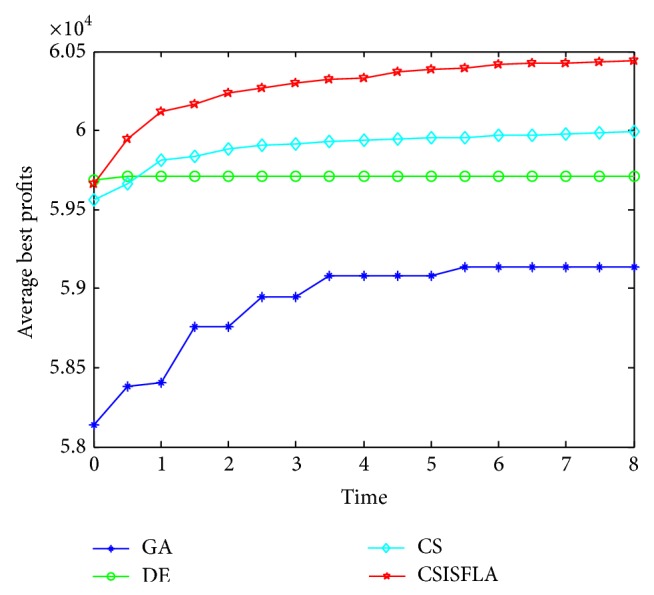
The convergence graphs of KP_7_.

**Figure 15:**
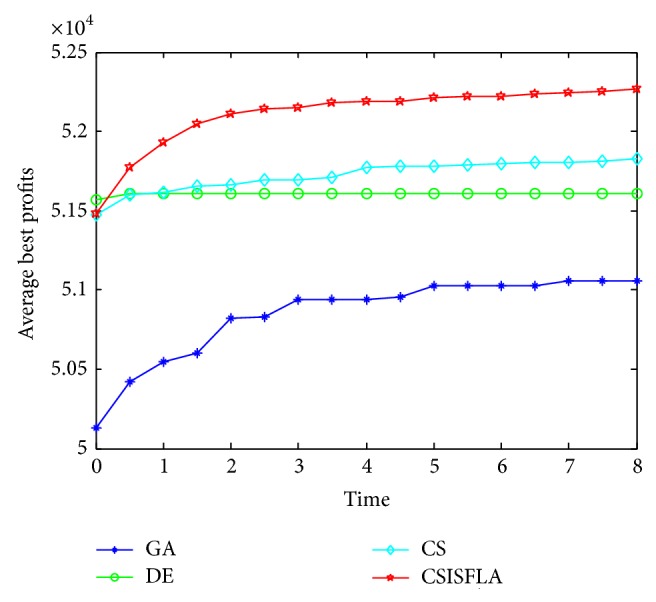
The convergence graphs of KP_14_.

**Figure 16:**
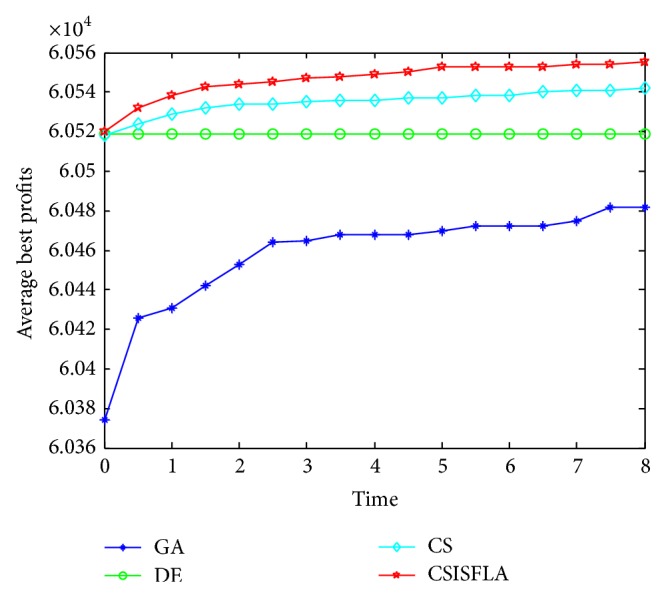
The convergence graphs of KP_19_.

**Figure 17:**
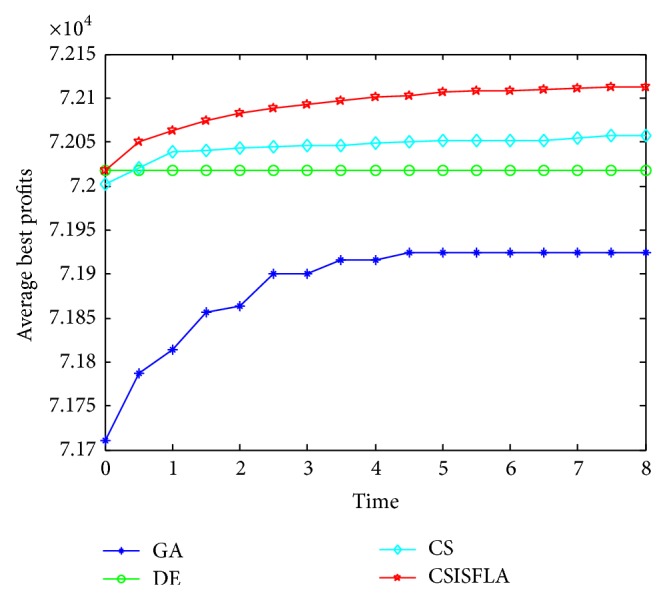
The convergence graphs of KP_24_.

**Figure 18:**
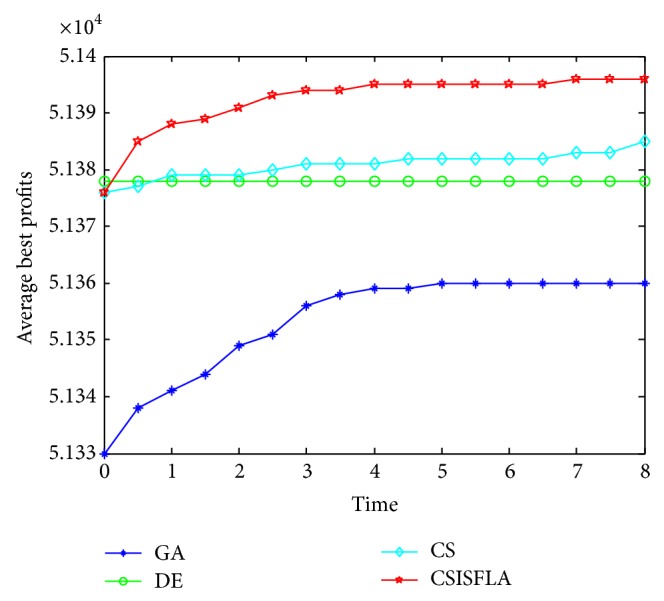
The convergence graphs of KP_29_.

**Figure 19:**
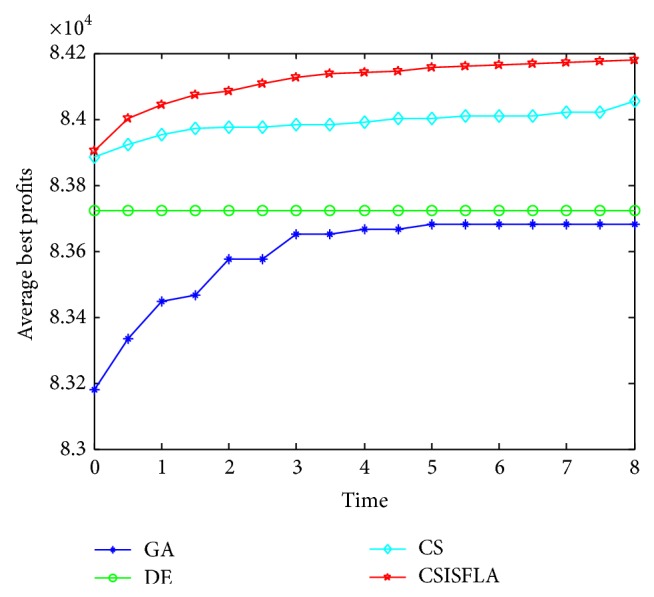
The convergence graphs of KP_34_.

We do experiment on 7 uncorrelated instances, 7 weakly correlated instances, and 5 other types of instances, respectively. The numerical results are given in Tables 6–11. The best values are emphasized in boldface. In addition, comparisons of the best profits obtained from the CSISFLA with those obtained from GA, DE, and CS for six KP instances with 1200 items are shown in Figures 8, 9, 10, 11, 12, and 13. Specifically, the convergence curves of four algorithms on six KP instances with 1200 items are also drawn in Figures 14, 15, 16, 17, 18, and 19. Through our careful observation, it can be analyzed as follows.

- Table 6 shows that CSISFLA outperforms GA, DE, and CS on almost all the uncorrelated knapsack instances in terms of computation accuracy and robustness. In particular, the best solution found by CSISFLA is slightly inferior to that obtained by DE on KP_3_. On closer inspection, “STD” is much smaller than that of the other algorithms except for KP_7_, which indicates the good stability of the CSISFLA and superior approximation ability.
- From Table 7, it can be seen that DE obtained the best, mean, and median results for the first four cases, and CS attained the best results for the last three cases. Although the optimal solutions obtained by the CSISFLA are worse than DE or CS, the CSISFLA obtained the worst, median, and STD results in KP_12_–KP_14_, which still can indicate that the CSISFLA has better stability. Above all, the well-known NFL theorem [52] has stated clearly that there is no heuristic algorithm best suited for solving all optimization problems. Unfortunately, although weakly correlated knapsack problems are closer to the real world situations [49], the CSISFLA does not appear clearly superior to the other two algorithms in solving such knapsack problems.
- Obviously, in point of search accuracy and convergence speed, it can be seen from Table 8 that CSISFLA outperforms GA, DE, and CS on all five strongly correlated knapsack problems. If anything, the STD values tell us that CSISFLA is only inferior to CS.
- Similar results were found from Tables 9, 10, and 11 and it can be inferred that CSISFLA can easily yield superior results compared with GA, DE, and CS. The series of experimental results confirm convincingly the superiority and effectiveness of CSISFLA.
- Figures 8–13 show a comparison of the best profits obtained by the four algorithms for six types of 1200 items.
- Figures 14–19 illustrate the average convergence curves of all the algorithms in 30 runs where we can observe that CS and CSISFLA usually show the almost same starting point. However, CSISFLA surpasses CS in point of the accuracy and convergence speed. CS performs the second best in hitting the optimum. DE shows premature phenomenon in the evolution and does not offer satisfactory performance along with the extending of the problem.

Based on previous analyses, we can draw a conclusion that the superiority of CSISFLA over GA, DE, and CS in solving six types of KP instances is quite indubitable. In general, CS is slightly inferior to CSISFLA, so the next best is CS. DE and GA perform the third-best and the fourth-best, respectively.

## 5. Conclusions

In this paper, we proposed a novel hybrid cuckoo search algorithm with improved shuffled frog-leaping algorithm, called CSISFLA, for solving 0-1 knapsack problems. Compared with the basic CS algorithm, the improvement of CSISFLA has several advantages. First, we specially designed an improved frog-leap operator, which not only retains the effect of the global optimal information on the frog leaping but also strengthens information exchange between frog individuals. Additionally, new individuals randomly generated with mutation rate. Second, we presented a novel CS model which is in an excellent combination with the rapid exploration of the global search space by Lévy flight and the fine exploitation of the local region by frog-leap operator. Third, CSISFLA employs hybrid encoding scheme; that is, to say, it conducts active searches in continuous real space, while the consequences are used to constitute the new solution in the binary space. Fourth, CSISFLA uses an effective GTM to assure the feasibility of solutions. The computational results show that CSISFLA outperforms the GA, DE, and CS in solution quality. Further, compared with ICS [26], the CSISFLA can be regarded as a combination of several algorithms and secondly the KP instances are more complex. The future work is to design more effective CS method for solving complex 0-1 KP and to apply the hybrid CS for solving other kinds of combinatorial optimization problems, multidimensional knapsack problem (MKP), and traveling salesman problem (TSP).

## Figures and Tables

**Algorithm 1:**
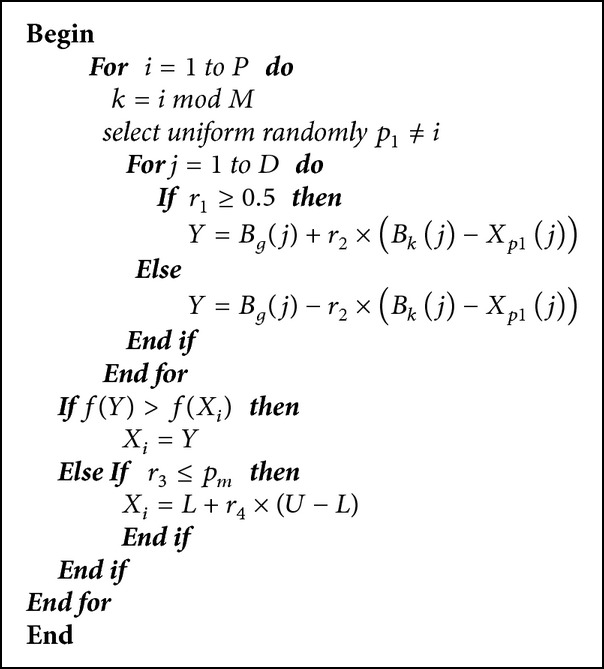
Improved shuffled frog-leaping algorithm.

**Algorithm 2:**
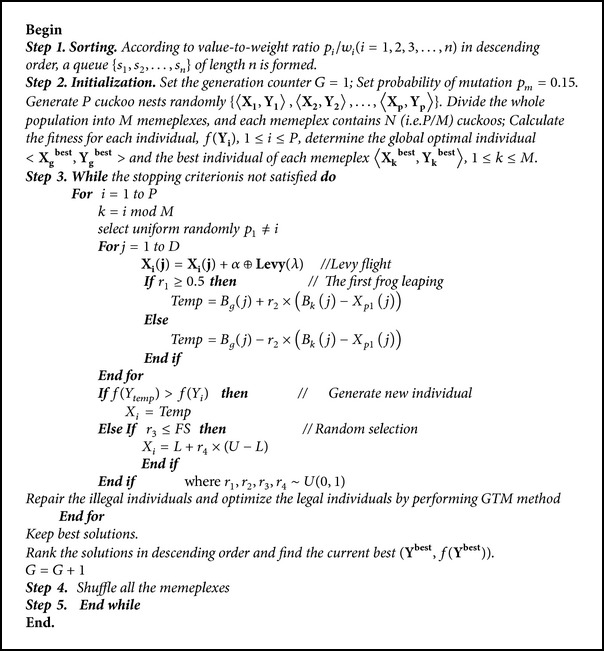
The main procedure of CSISFLA algorithm.

**Table 1:** Representation of population in CSISFLA.

〈**X**_1_, **B**_1_〉	〈**X**_2_, **B**_2_〉	⋯	〈**X**_*i*_, **B**_*i*_〉	⋯	〈**X**_*n*_, **B**_*n*_〉

**Table 2:** Knapsack problem instances.

Problem	Correlation	Dimension	Target weight	Total weight	Total values
KP_1_	Uncorrelated	150	6471	8628	8111
KP_2_	Uncorrelated	200	8328	11104	10865
KP_3_	Uncorrelated	300	12383	16511	16630
KP_4_	Uncorrelated	500	20363	27150	28705
KP_5_	Uncorrelated	800	33367	44489	44005
KP_6_	Uncorrelated	1000	41948	55930	54764
KP_7_	Uncorrelated	1200	49485	65980	66816
KP_8_	Weakly correlated	150	6403	8538	8504
KP_9_	Weakly correlated	200	8358	11144	11051
KP_10_	Weakly correlated	300	12554	16739	16778
KP_11_	Weakly correlated	500	20758	27677	27821
KP_12_	Weakly correlated	800	33367	44489	44491
KP_13_	Weakly correlated	1000	41849	55799	55683
KP_14_	Weakly correlated	1200	49808	66411	56811
KP_15_	Strongly correlated	300	12247	16329	19329
KP_16_	Strongly correlated	500	21305	28407	33406
KP_17_	Strongly correlated	800	33367	44489	52489
KP_18_	Strongly correlated	1000	40883	54511	64510
KP_19_	Strongly correlated	1200	50430	67240	79240
KP_20_	Multiple strongly correlated	300	12908	17211	23651
KP_21_	Multiple strongly correlated	500	20259	27012	37903
KP_22_	Multiple strongly correlated	800	32767	43689	61140
KP_23_	Multiple strongly correlated	1000	42442	56589	77940
KP_24_	Multiple strongly correlated	1200	50222	66963	92653
KP_25_	Profit ceiling	300	12666	16888	17181
KP_26_	Profit ceiling	500	19811	26415	26913
KP_27_	Profit ceiling	800	32011	42681	43497
KP_28_	Profit ceiling	1000	42253	56337	57381
KP_29_	Profit ceiling	1200	50208	66944	68157
KP_30_	Circle	300	12554	16739	26448
KP_31_	Circle	500	20812	27749	43880
KP_32_	Circle	800	32581	43441	69527
KP_33_	Circle	1000	42107	56143	88220
KP_34_	Circle	1200	49220	65627	104287

**Table 3:** The effect of *M* and *N* on the performance of the CSISFLA.

Instance	*N*	*M* = 2				*M*	*N* = 10			
		Best	Worst	Mean	STD		Best	Worst	Mean	STD
KP_9_	10	8727	**8704**	8711	5.5	2	**8727**	8704	8711	5.5
	15	**8728**	8701	**8715**	**6.8**	3	8725	8701	8713	7.0
	20	**8730**	8702	**8718**	6.5	4	8726	**8708**	8717	**6.3**

KP_10_	10	13152	13124	13140	8.7	2	13152	13124	13140	8.7
	15	**13168**	13120	13144	12.6	3	13167	**13131**	**13146**	**8.2**
	20	**13174**	13126	**13148**	13.3	4	**13168**	**13128**	**13148**	**9.4**

KP_11_	10	21820	21737	21773	22.1	2	21820	21737	21773	22.1
	15	21827	**21756**	**21786**	**17.3**	3	**21840**	21735	21783	24.6
	20	21814	**21757**	21778	**15.4**	4	**21848**	21742	**21788**	23.5

**Table 4:** The effect of *p*_*m*_ on the performance of the CSISFLA.

Instance	0	0.05	0.1	0.15	0.2	0.3	0.4	0.5	0.6	0.7	0.8	0.9	1.0
KP_1_													
Best	7474	**7475**	**7475**	**7475**	**7475**	7474	**7475**	7474	7474	7474	7473	7474	7459
Worst	7430	7469	7468	**7471**	**7471**	7463	7457	7451	7451	7446	7437	7427	7407
Mean	7461	7473	**7474**	**7474**	7473	7471	7470	7468	7468	7461	7455	7448	7436
STD	12.60	1.50	1.57	**0.93**	1.27	3.57	4.96	6.03	5.87	8.83	10.11	11.17	13.88
KP_2_													
Best	**9865**	**9865**	**9865**	**9865**	9863	9864	9860	9859	9850	9847	9844	9843	9842
Worst	9821	**9847**	9845	9844	9839	9823	9830	9818	9804	9778	9775	9768	9757
Mean	9847	**9858**	9856	9857	9852	9848	9847	9841	9833	9830	9812	9810	9783
STD	11.96	5.75	6.12	**5.32**	6.84	10.60	7.99	11.89	12.35	16.86	21.92	21.12	20.24
KP_8_													
Best	6676	6674	6673	6672	6671	6672	6672	6671	**6678**	6666	6666	6662	6654
Worst	6658	6662	6663	**6665**	6662	6663	6662	6657	6655	6650	6652	6645	6642
Mean	6668	**6671**	6669	6669	6668	6668	6668	6664	6664	6659	6658	6652	6647
STD	4.59	2.95	2.59	**2.04**	2.44	2.79	2.39	4.17	4.45	4.06	3.88	4.27	3.17
KP_9_													
Best	8730	**8734**	**8734**	8728	8731	8720	8723	8716	8712	8710	8707	8701	8688
Worst	**8707**	8703	8705	8701	8700	8702	8695	8684	8682	8675	8677	8664	8655
Mean	8716	**8718**	**8718**	8715	8714	8711	8707	8702	8697	8693	8690	8682	8676
STD	6.23	8.79	6.66	6.85	7.45	**4.59**	7.20	7.97	7.50	9.75	7.27	10.06	7.76

**Table 5:** Parameter settings of GA, DE, CS, and CSISFLA on 0-1 knapsack problems.

Algorithm	Parameter	Value
GA [41]	*Population size *	100
	*Crossover probability *	0.6
	*Mutation probability *	0.001
DE [42, 43]	*Population size *	100
	*Crossover probability *	0.9
	*Amplification factor *	0.3
CS [24]	*Population size *	40
	*p*_*a*_	0.25
CSISFLA	*M*	4
	*N*	10
	*p*_*m*_	0.15

**Table 6:** Experimental results of four algorithms with uncorrelated KP instances.

Instance	Algorithm	Best	Worst	Mean	Median	STD
KP_1_	GA	7316	6978	7200	7208	75.78
	DE	**7475**	7433	7471	7473	7.68
	CS	7472	7358	7403	7405	27.82
	CSISFLA	**7475**	**7467**	**7473**	**7474**	**1.56**

KP_2_	GA	9673	9227	9503	9507	97.39
	DE	**9865**	9751	9854	**9865**	22.52
	CS	9848	9678	9737	9734	33.22
	CSISFLA	**9865**	**9837**	**9856**	9858	**7.23**

KP_3_	GA	15022	14275	14756	14795	158.91
	DE	**15334**	15088	15287	15301	54.45
	CS	15224	15024	15092	15081	51.37
	CSISFLA	15327	**15248**	**15297**	**15302**	**18.48**

KP_4_	GA	25882	25212	25498	25493	150.68
	DE	26333	25751	26099	26096	135.88
	CS	26208	25786	25936	25911	103.4
	CSISFLA	**26360**	**26193**	**26284**	**26277**	**38.54**

KP_5_	GA	39528	38462	38976	39014	243.62
	DE	39652	39215	39410	39399	113.28
	CS	40223	39416	39565	39514	179.98
	CSISFLA	**40290**	**39885**	**40072**	**40081**	**91.97**

KP_6_	GA	49072	47835	48483	48570	316.62
	DE	49246	48835	48989	48979	101.11
	CS	49767	49024	49164	49142	143.08
	CSISFLA	**49893**	**49567**	**49744**	**49737**	**97.52**

KP_7_	GA	59793	58351	59135	59225	370.86
	DE	59932	59488	59707	59727	**110.39**
	CS	60629	59708	59939	59884	166.43
	CSISFLA	**60779**	**60264**	**60443**	**60420**	130.56

**Table 7:** Experimental results of four algorithms with weakly correlated KP instances.

Instance	Algorithm	Best	Worst	Mean	Median	STD
KP_8_	GA	6627	6531	6593	6593	20.63
	DE	**6676**	6657	**6674**	**6676**	4.80
	CS	6660	6637	6648	6646	6.79
	CSISFLA	6673	**6663**	6668	6668	**2.23**

KP_9_	GA	8658	8501	8588	8590	33.38
	DE	**8743**	**8743**	**8743**	**8743**	**0.00**
	CS	8717	8644	8676	8671	18.23
	CSISFLA	8728	8701	8714	8714	6.87

KP_10_	GA	13062	12939	12997	12991	30.64
	DE	**13202**	**13158**	**13186**	**13186**	**9.76**
	CS	13157	13069	13094	13087	21.91
	CSISFLA	13168	13120	13145	13145	11.90

KP_11_	GA	21671	21470	21571	21576	48.85
	DE	**21951**	21745	**21858**	**21859**	37.61
	CS	21935	21670	21746	21722	76.53
	CSISFLA	21827	**21756**	21788	21787	**16.66**

KP_12_	GA	34587	34314	34488	34499	63.23
	DE	34814	34578	34721	34718	64.50
	CS	**34987**	34621	34697	34654	100.38
	CSISFLA	34818	**34721**	**34760**	**34758**	**22.87**

KP_13_	GA	43241	42938	43082	43073	75.51
	DE	43327	43162	43217	43211	43.64
	CS	**43737**	43216	43340	43264	166.53
	CSISFLA	43409	**43312**	**43367**	**43368**	**27.23**

KP_14_	GA	51472	50414	51058	51135	265.56
	DE	51947	51444	51600	51569	108.83
	CS	**53333**	51601	51831	51788	299.35
	CSISFLA	52403	**52077**	**52267**	**52264**	**86.19**

**Table 8:** Experimental results of four algorithms with strongly correlated KP instances.

Instance	Algorithm	Best	Worst	Mean	Median	STD
KP_15_	GA	14785	14692	14754	14762	25.93
	DE	14797	14781	14789	14787	4.90
	CS	14804	14791	14797	**14797**	**2.43**
	CSISFLA	**14807**	**14795**	**14798**	**14797**	3.46

KP_16_	GA	25486	25402	25458	25465	21.61
	DE	25502	25481	25492	25493	4.21
	CS	25514	25502	25506	25505	**3.49**
	CSISFLA	**25515**	**25505**	**25510**	**25512**	3.94

KP_17_	GA	40087	39975	40039	40041	28.33
	DE	40111	40068	40089	40088	8.66
	CS	40107	40096	40103	40105	**3.88**
	CSISFLA	**40117**	**40098**	**40111**	**40113**	5.12

KP_18_	GA	49332	49225	49300	49309	27.26
	DE	49363	49333	49346	49345	7.50
	CS	49380	49350	49364	49363	**7.04**
	CSISFLA	**49393**	**49362**	**49373**	**49373**	7.90

KP_19_	GA	60520	60418	60482	60489	26.62
	DE	60540	60501	60519	60519	8.55
	CS	60558	60530	60542	60540	6.77
	CSISFLA	**60562**	**60539**	**60549**	**60550**	**5.70**

**Table 9:** Experimental results of four algorithms with multiple strongly correlated KP instances.

Instance	Algorithm	Best	Worst	Mean	Median	STD
KP_20_	GA	18346	18172	18284	18288	38.39
	DE	18387	18335	18354	18348	15.25
	CS	18386	18355	18368	18368	**4.73**
	CSISFLA	**18388**	**18368**	**18381**	**18386**	8.03

KP_21_	GA	29525	29387	29461	29462	31.97
	DE	29548	29488	29519	29520	14.10
	CS	29589	29527	29555	29549	13.94
	CSISFLA	**29609**	**29562**	**29581**	**29585**	**12.38**

KP_22_	GA	47645	47494	47568	47575	39.72
	DE	47704	47620	47659	47657	20.68
	CS	47727	47673	47696	47695	15.09
	CSISFLA	**47757**	**47697**	**47732**	**47736**	**13.02**

KP_23_	GA	60529	60312	60455	60463	47.39
	DE	60572	60508	60534	60530	**13.98**
	CS	60607	60540	60576	60574	16.96
	CSISFLA	**60650**	**60579**	**60615**	**60612**	15.75

KP_24_	GA	72063	71725	71914	71917	64.42
	DE	72072	71973	72018	72018	19.38
	CS	72094	72031	72058	72057	**15.93**
	CSISFLA	**72151**	**72070**	**72112**	**72111**	21.20

**Table 10:** Experimental results of four algorithms with profit ceiling KP instances.

Instance	Algorithm	Best	Worst	Mean	Median	STD
KP_25_	GA	12957	12948	12955	12957	2.53
	DE	12957	12951	12953	12954	1.83
	CS	12957	12954	12957	12957	0.76
	CSISFLA	**12957**	**12957**	**12957**	**12957**	**0.00**

KP_26_	GA	20295	20268	20285	20286	7.37
	DE	20301	20292	20294	20294	2.17
	CS	20304	20295	20299	20298	**1.86**
	CSISFLA	**20307**	**20298**	**20304**	**20304**	2.28

KP_27_	GA	32796	32769	32785	32787	6.99
	DE	32802	32793	32797	32796	**2.63**
	CS	32811	32799	32803	32802	3.12
	CSISFLA	**32820**	**32808**	**32812**	**32811**	3.34

KP_28_	GA	43248	43215	43234	43236	8.76
	DE	43257	43245	43249	43248	3.57
	CS	43269	43251	43257	43254	4.41
	CSISFLA	**43272**	**43260**	**43266**	**43266**	**2.88**

KP_29_	GA	51378	51348	51364	51366	7.25
	DE	51384	51372	51378	51378	**3.04**
	CS	**51399**	51378	51385	51384	4.32
	CSISFLA	**51399**	**51390**	**51396**	**51396**	3.10

**Table 11:** Experimental results of four algorithms with circle KP instances.

Instance	Algorithm	Best	Worst	Mean	Median	STD
KP_30_	GA	21194	20899	21086	21096	71.44
	DE	21333	21192	21264	21277	32.46
	CS	21333	21194	21261	21261	**18.57**
	CSISFLA	**21333**	**21263**	**21300**	**21295**	34.04

KP_31_	GA	35262	34982	35112	35124	82.25
	DE	35343	35184	35247	35267	38.08
	CS	35345	35271	35297	35277	31.29
	CSISFLA	**35414**	**35342**	**35354**	**35345**	**23.23**

KP_32_	GA	55976	55451	55746	55771	116.83
	DE	56063	55914	55964	55954	44.95
	CS	**56280**	55988	56057	56061	55.01
	CSISFLA	56273	**56130**	**56185**	**56201**	**38.65**

KP_33_	GA	70739	70247	70487	70456	113.53
	DE	70806	70641	70696	70684	**38.21**
	CS	70915	70729	70789	70797	42.50
	CSISFLA	**71008**	**70867**	**70924**	**70939**	41.17

KP_34_	GA	83969	83339	83723	83757	142.75
	DE	84040	83820	83912	83899	56.64
	CS	**84645**	83954	84055	84033	121.94
	CSISFLA	84244	**84099**	**84175**	**84181**	**38.36**
